# Decomposing Current Mortality Differences Into Initial Differences and Differences in Trends: The Contour Decomposition Method

**DOI:** 10.1007/s13524-017-0599-6

**Published:** 2017-07-28

**Authors:** Dmitri A. Jdanov, Vladimir M. Shkolnikov, Alyson A. van Raalte, Evgeny M. Andreev

**Affiliations:** 1 Max Planck Institute for Demographic Research, Konrad Zuse Str. 1, 18057 Rostock, Germany; 2 National Research University Higher School of Economics, Bol’shoj Trehsvjatitel’skij pereulok, 3, 109028 Moscow, Russia

**Keywords:** Decomposition, Demographic change, Stepwise replacement, Mortality, Aggregate demographic measure

## Abstract

This study proposes a new decomposition method that permits a difference in an aggregate measure at a final time point to be split into additive components corresponding to the initial differences in the event rates of the measure and differences in trends in these underlying event rates. For instance, when studying divergence in life expectancy, this method allows researchers to more easily contrast age-specific mortality trends between populations by controlling for initial age-specific mortality differences. Two approaches are assessed: (1) an additive change method that uses logic similar to cause-of-death decomposition, and (2) a contour decomposition method that extends the stepwise replacement algorithm along an age-period demographic contour. The two approaches produce similar results, but the contour method is more widely applicable. We provide a full description of the contour replacement method and examples of its application to life expectancy and lifetime disparity differences between the United States and England and Wales in the period 1980–2010.

**Electronic supplementary material:**

The online version of this article (doi:10.1007/s13524-017-0599-6) contains supplementary material, which is available to authorized users.

## Introduction

In a prior study, the mean length of life and the lifetime disparity (a measure of variation in ages at death also known as *lifetime losses* or *e* ^†^) in the United States were the compared with corresponding quantities in other advanced countries (Shkolnikov et al. 2011). The United States was found to experience outstandingly high lifetime losses. A comparison with England and Wales was especially intriguing because the two Anglo-Saxon countries did not significantly differ from each other in terms of life expectancy before the mid-1980s. Over the last 25 years, the formerly minor Anglo-American life expectancy gap has widened. For the last 50 years, the lifetime disparity in the United States has been consistently and substantially higher than that in England and Wales.

Conventional decomposition analysis showed that the life expectancy and the lifetime disparity differences between England and Wales and the United States in the early 2000s were determined by higher American mortality at infant, young adult, and midlife ages, combined with lower mortality at old ages. In addition to this decomposition outcome, one may be interested in determining the extent to which the intercountry difference of today is a legacy of past age-specific differences and the extent to which it is a result of differences in age-specific mortality trends.

At first, splitting a cross-sectional difference according to the initial difference and the trend looks straightforward. One can consider three independent decompositions: at the initial time point between the two populations, and the longitudinal decompositions between the initial and the final time points for each of the two populations. There is, however, a difficulty generally related to the nonlinearity of the functions being decomposed, such as life expectancy or lifetime disparity. For any age group, the respective age component of the decomposition of the difference between the two populations at the final time point cannot be obtained by summation of the age components from the three independent decompositions. The following empirical example illustrates this further.

Using the conventional method of decomposition (Andreev 1982; Arriaga 1984; Pressat 1985), one finds that the age group 40–59 in 2010 contributed 0.92 years to the total difference between male life expectancies in England and Wales and in the United States. Earlier, in 1980, this value was equal to 0.50 years. One might think that 0.50 years is the contribution of the initial conditions to the age 40–59 component of the Anglo-American gap in 2010 and that the trend component adds 0.42 (= 0.92 – 0.50) years. On the other hand, it is equally possible to assume that the contribution of the trend should be equal to the age 40–59 component of the life expectancy increase from 1980 to 2010 in England and Wales minus the corresponding component of the life expectancy increase in the United States from 1980 to 2010. However, decompositions of these two increases return the 40–59 component equal to 1.59 years for England and Wales and 1.29 years for the United States, with their difference being 0.30 years—a value that is substantially smaller than 0.42 years.

The discrepancy is induced by the nonlinearity of the life expectancy as a function of age-specific death rates. The latter leads to a side influence of mortality contexts (death rates at ages other than *x*) on the age component of a chosen age *x*, illustrating a need for an adjustment of age components for the differences between the mortality contexts.

The implication is that at different time points, contributions of a chosen age *x* to the interpopulation difference in life expectancy are incomparable due to temporal change in the mortality contexts. In particular, contributions of the initial between-population differences in death rates to the initial life expectancy gap are not equivalent to contributions of these initial differences between death rates to the life expectancy gap at a final time point. Therefore, contributions at the starting time point should be adjusted somehow to the new mortality regime with (typically) lower death rates.

Here we propose a decomposition method to overcome this difficulty and to quantify correctly the relative importance of the past conditions and the temporal change to a contemporary difference in an aggregate index between two populations in question. In particular, this method allows us to assess the effect of recent factor-specific trends (e.g., age) on variations in an aggregate index while controlling for initial factor-specific differences. The method splits the age components of a contemporary difference into partitions produced by the initial mortality differences between the two populations (initial age components) and mortality trends in the two populations (trend age components). For this additional splitting, we use a new algorithm of contour replacement, which is essentially an extension of the earlier algorithm of stepwise replacement. In what follows, we describe the latter algorithm and situate it within a more general decomposition agenda.

For the sake of simplicity, we consider a one-dimensional decomposition by age only. For the whole decomposition methodology, decomposition by age is central. If one knows how to decompose a difference in the aggregate index by age, further splitting components by cause of death, birth order, or population subgroup is usually a simpler problem. We consider only mortality and length of life, but the same methodology would be applicable to fertility or migration.

### Decomposition Agenda and the Stepwise Replacement Algorithm

Researchers often compare populations by the value of an aggregated scalar index: for instance, life expectancy at birth or an index of lifespan variation. These indices themselves are functions of independent covariates, such as event rates across various dimensions, including age, cause of death, and population group. The classic decomposition task is to attribute the total between-population difference in the aggregate index to contributions from differences in the covariates. Demographers are mostly dealing with additive decompositions, which assume that a sum of covariate contributions is equal to the total difference, even though the index itself is a nonlinear function.

In 1955, Kitagawa proposed the first decomposition method to gain insight into differences in crude death rates (CDR) between populations. Noting that the CDR was a population-weighted mean of age-specific death rates, she proposed a simple formula to compute age-specific components of the total difference split into mortality and population-composition partitions. In the early 1990s, Das Gupta (1991, 1994) proposed a generalization of this decomposition method for a multidimensional case. The aggregate index had to be a linear function of the covariates.

Nonlinearity obviously complicates the decomposition problem because for a nonlinear dependent index *f*(*u*), the first partial derivative ∂*f* / ∂*u* depends on *u*. In the 1980s, four researchers working independently deduced formulae for the decomposition of a difference between two life expectancy values by age (Andreev 1982; Arriaga 1984; Pollard 1982; Pressat 1985). Although the formulae by Andreev, Arriaga, and Pressat are equivalent and return the same age components, age components resulting from Pollard’s formula are slightly different (Shkolnikov et al. 2001). Because life expectancy is a highly nonlinear aggregate function of age-specific death rates, decompositions of life expectancy differences are nonsymmetrical and nontransitive (path dependent) with respect to populations and years being compared (Horiuchi et al. 2008).

In all these life expectancy decomposition approaches of the 1980s, the derivation of analytical expressions for the age components was based on particular properties of life expectancy as a mathematical construct. The 2000s saw growing interest in decomposition of life table–based measures of dispersion, such as the Gini coefficient, standard deviation, lifetime disparity (*e* ^†^), variance, and life table entropy (Edwards and Tuljapurkar 2005; Gillespie et al. 2014; Nau and Firebaugh 2012; Shkolnikov et al. 2003; van Raalte and Caswell 2013; Zhang and Vaupel 2009).

What if one wanted to decompose an index other than life expectancy, with a different mathematical construct, for which analytical expressions for components could not be derived? To address this question, researchers worked on general (universal) decomposition methods that allowed the numerical decomposition of a difference in values of any aggregate index. So far, two methods have been developed: a model of continuous change, and a discrete algorithm of stepwise replacement. The *continuous change method* models the total change between two time points or two different populations as a sequence of small steps with the effects of age-specific rates being computed by the numerical integration of partial derivatives of the index function with respect to the corresponding age-specific rates (Horiuchi et al. 2008). A closely related but independently developed framework is based on an absorbing Markov chain formulation of the life table and matrix calculus (see Caswell 2001: chapter 10). The second general method, the *stepwise replacement algorithm* (Andreev et al. 2002), extends Das Gupta’s logic by presenting the total change in the dependent index as a sum of effects of the sequential replacement of age-specific rates progressing from age 0 to the highest age (see Online Resource 1 for a summary of the method). This order of replacement of the age-specific death rates guarantees that in the case of life expectancy, the resulting age components are exactly the same as those calculated according to the earlier (and most commonly used) decomposition formula by Arriaga-Andreev-Pressat (Andreev et al. 2002).

### Flow of the Study

We consider first a simple and straightforward method of *additive change*, which can be applied only if certain conditions are fulfilled. We then develop a more complicated method of unrestricted applicability, which we term the *contour decomposition method*. We make an extensive comparison of decomposition results using the two methods on data from the Human Mortality Database (HMD n.d.) and show that in the *additive change* variant, empirical applicability is limited by frequent violation of the required conditions. Finally, we return to the example of the contrast between the United States and England and Wales and use the newly developed decomposition methodology to assess the importance of initial conditions and mortality trends for the differences between the two countries in the mean length of life and in the lifetime disparity.

The list of mathematical abbreviations is given in Table 4 in the appendix.

## Methods

### Decomposition Task

**Fig. 1:**
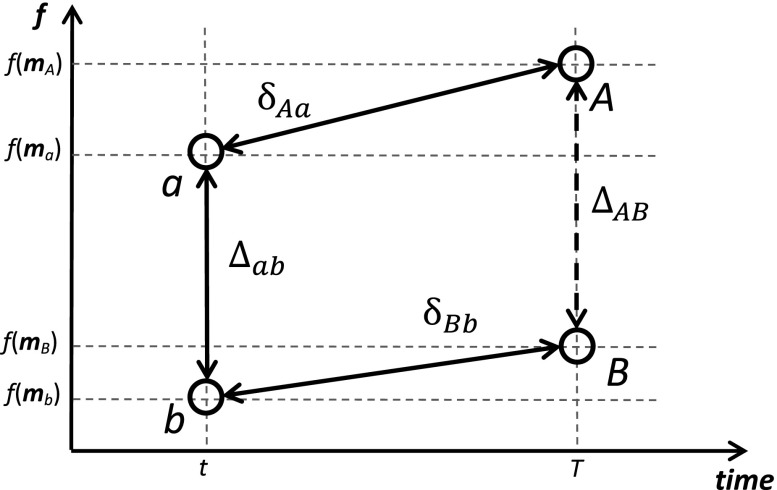
Cross-sectional differences and longitudinal changes in an aggregate demographic measure in two populations

Imagine that an aggregate measure (say, life expectancy) in two populations is measured at two time points (Fig. 1). Clearly, the between-population difference at the second time point *T* depends on both the initial age-specific mortality differences at time point *t*, and on changes in age-specific mortality between *t* and *T*.

Let us formally define the decomposition problem. Assume that the demographic measure of interest *f*(.) for a population *A* is a function of a vector of age-specific event rates:

$$ E=f\left({\mathbf{m}}_A\right), $$

where **m**_*A*_ = [*m*_*A*_(*x*_1_), . . . , *m*_*A*_(*x*_*i*_), . . . , *m*_*A*_(*x*_*n*_)].^1^

For the two populations *A* and *B* at time *T*, measure *E* has values *f*(**m**_*A*_) and *f*(**m**_*B*_), respectively (Fig. 1). The final between-population difference is

1 $$ {\Delta}_{AB}=f\left({\mathbf{m}}_A\right)-f\left({\mathbf{m}}_B\right). $$

This difference can be decomposed by age

2 $$ {\Delta}_{AB}={\sum}_{i=1}^n{\Delta}_{AB}^i. $$

In Eq. (2), the age components can be computed using different methods of decomposition. The age component pertaining to age *i* reflects the contribution of the between-population mortality difference *m*_*A*_(*x*_*i*_) − *m*_*B*_(*x*_*i*_) at time *T* to the total difference ∆_*AB*_.

At the same time, the age component *i* at time *T* can be considered to have resulted from the initial mortality difference at time *t* and age *i* between the two populations *m*_*a*_(*x*_*i*_) − *m*_*b*_(*x*_*i*_) and the uneven temporal mortality changes in the two populations *m*_*A*_(*x*_*i*_) − *m*_*a*_(*x*_*i*_) and *m*_*B*_(*x*_*i*_) − *m*_*b*_(*x*_*i*_). Thus, the *decomposition task* is to split the final difference Δ_*AB*_ into age-specific contributions produced by the initial between-population difference in the age-specific rates (initial component) and contributions due to different (within-population) age-specific mortality trends (trend component). The primary requirement is that at every age, the sum of the initial and the trend components is equal to the total age-specific component from Eq. (2):

3 $$ {\Delta}_{AB}={\sum}_{i=1}^n\left({Initial}^i+{Trend}^i\right)={\sum}_{i=1}^n\left({\Delta}_{ab\mid AB}^i+{\updelta}_{ab\mid AB}^i\right) $$

4 $$ {Initial}^i+{Trend}^i={\Delta}_{ab\mid AB}^i+{\updelta}_{ab\mid AB}^i={\Delta}_{AB}^i,i=1,\dots, n, $$

where ${\Delta}_{ab\mid AB}^i$ and ${\updelta}_{ab\mid AB}^i$ denote initial and trend components, respectively.^2^

### Between-Population and Within-Population Decompositions

Let us first perform the age decomposition of the between-population difference at time *T* (Eq. (2)). For this decomposition, familiar from earlier studies, we apply the stepwise replacement algorithm. This technique estimates the age-specific contributions to the change in a function such as (for example) life expectancy or lifetime disparity as changes in the value of this function produced by stepwise replacement of the underlying rates, *m*_*A*_(*x*_*i*_) → *m*_*B*_(*x*_*i*_) (for summary of the method, see Online Resource 1). This is obviously an exact decomposition when *f* is linear with respect to the age-specific rates. When *f* is nonlinear, the order of replacement influences the conditional effects estimated for each age, but respective differences are minor (Andreev et al. 2002; Horiuchi et al. 2008). As with any other decomposition technique, the stepwise replacement provides an approximate estimate of the influence of every age group for nonlinear dependent indices. The algorithm calculates a series of effects conditional on the replacement order. As we mentioned earlier, it is sensible to use the ascending sequence of ages from 0 to the highest age for consistency with earlier life expectancy decomposition formulae.

The key step in the stepwise replacement algorithm is replacement of age-specific rates in the vector **m**. We denote ${\mathbf{m}}_{AB}^{\left[i\right]}$ as the vector of age-specific rates in population *B* after replacement of *i* first elements of this vector by corresponding age-specific rates in population *A*:

5 $$ {\mathbf{m}}_{AB}^{\left[i\ \right]}=\left[{m}_A\left({x}_1\right),\dots, {m}_A\left({x}_i\right),{m}_B\left({x}_{i+1}\right),\dots, {m}_B\left({x}_n\right)\right]\kern0.5em \mathrm{with}\kern0.5em {\mathbf{m}}_{AB}^{\left[0\right]}={\mathbf{m}}_B. $$

According to Andreev et al. (2002), the component of the total difference Δ_*AB*_ produced by the elementary difference between *A* and *B* in event rates at age *x*_*i*_ is

6 $$ {\Delta}_{AB}^i=f\left({\mathbf{m}}_{AB}^{\left[i\right]}\right)-f\left({\mathbf{m}}_{AB}^{\left[i-1\right]}\right),i=1,\dots i\dots, n. $$

The basic equation of the stepwise replacement algorithm for decomposition Eq. (2) of the between-population difference Δ_*AB*_ is

7 $$ f\left({\mathbf{m}}_A\right)-f\left({\mathbf{m}}_B\right)={\sum}_{i=1}^n\left[f\left({\mathbf{m}}_{AB}^{\left[i\right]}\right)-f\left({\mathbf{m}}_{AB}^{\left[i-1\right]}\right)\right]={\sum}_{i=1}^n{\Delta}_{AB}^i. $$

Equation (7) reflects the process of replacement of elements of vector **m**_*B*_ by elements of vector **m**_*A*_. Because there is no preference for the direction of the replacement (*A*→*B* or *B*→*A*), the final age-specific components are calculated as an average:

8 $$ {\varDelta}_{AB}={\sum}_{i=1}^n\frac{1}{2}\ \left({\Delta}_{AB}^i-{\Delta}_{BA}^i\right). $$

Obviously, decomposition of the between-population difference ∆_*ab*_ at time *t* (Fig. 1) can be carried out with Eqs. (6)–(8) applied to the populations *a* and *b* instead of *A* and *B*.

The same way, one can decompose temporal changes within populations δ_*Aa*_ and δ_*Bb*_ (Fig. 1). Equations similar to Eqs. (6)–(8) for the trend decomposition of the change δ_*Aa*_ are

9 $$ {\updelta}_{Aa}^i=f\left({\mathbf{m}}_{Aa}^{\left[i\right]}\right)-f\left({\mathbf{m}}_{Aa}^{\left[i-1\right]}\right),\kern0.75em i=1,\dots, n $$

10 $$ f\left({\mathbf{m}}_A\right)-f\left({\mathbf{m}}_a\right)={\sum}_{i=1}^n\left[f\left({\mathbf{m}}_{Aa}^{\left[i\right]}\right)-f\left({\mathbf{m}}_{Aa}^{\left[i-1\right]}\right)\right]={\sum}_{i=1}^n{\updelta}_{Aa}^i $$

11 $$ {\updelta}_{Aa}={\sum}_{i=1}^n\frac{1}{2}\ \left({\updelta}_{Aa}^i-{\updelta}_{aA}^i\right). $$

At first glance, it seems that the target decomposition in Eqs. (3)–(4) can be obtained by combining between-population and within-population decompositions in Eqs. (6)–(8) and (9)–(11). Indeed, the following equation looks very much like Eq. (3):

$$ {\varDelta}_{AB}={\sum}_{i=1}^n{\varDelta}_{ab}^i+{\sum}_{i=1}^n\left({\updelta}_{Aa}^i-{\updelta}_{Bb}^i\right). $$

There is a problem however. As we mentioned earlier, within each age group *x*_*i*_ the between-population age component ${\Delta}_{AB}^i$ from Eq. (6) is not equal to the sum of the initial between-population component and the difference between the within-population components:

12 $$ {\varDelta}_{AB}^i\ \ne\ {\varDelta}_{ab}^i+\left({\updelta}_{Aa}^i-{\updelta}_{Bb}^i\right),i=1,2,\dots, n. $$

This inequality is a general and formal expression of the numerical contradiction in the Anglo-American decomposition example that we placed in the Introduction and is related to the nontransitivity (or path dependence) of decomposition outcomes for nonlinear indices (for more details, see Andreev et al. 2002; Horiuchi et al. 2008). It implies that the condition indicated by Eq. (4) is not fulfilled.

Hence, we have to admit that the target decomposition in Eqs. (3)–(4) cannot be expressed via two between-population decompositions in Eqs. (6)–(8) at times *t* and *T* and two trend decompositions in Eqs. (9)–(11) within the two populations.

In the next section, we show how the general algorithm of stepwise replacement can be extended for completing our decomposition task.

### Decomposition Methods

We present two alternative approaches to the target decomposition task in Eqs. (3)–(4): the additive change method, and the contour decomposition method. For reasons that will become clear, we argue in favor of the second method.

#### Additive Change Method

The simplest and intuitively transparent solution to the decomposition problem is as follows. Let us denote Δ**m**_*A*_ = **m**_*A*_ − **m**_*a*_. Obviously,

$$ {\Delta}_{AB}=f\left({\mathbf{m}}_a+\Delta {\mathbf{m}}_A\right) - f\left({\mathbf{m}}_b+\Delta {\mathbf{m}}_B\right). $$

In this equation, *f* depends on two vectors **m** and Δ**m**. The former reflects the initial conditions at time *t*, the latter reflects the trend. The decomposition task in this form is similar to the decomposition of life expectancy or other life table index by age and cause of death, where **m** and Δ**m** may be considered as two vectors of death rates for two fictitious causes of death. The age components are calculated by means of the standard stepwise replacement algorithm for decomposition by age and cause of death (Andreev et al. 2002). A detailed description of this approach is given in Online Resource 2.

However, unlike replacement of nonnegative death rates for causes of death, replacements of **m** and Δ**m** do not make sense in cases when, for some *i*, *m*_*a*_(*x*_*i*_) + ∆*m*_*A*_(*x*_*i*_) or *m*_*b*_(*x*_*i*_) + ∆*m*_*B*_(*x*_*i*_) have negative values. For some demographic indices *f*(.), this limitation can be relaxed (see Online Resource 2), but the main advantage of the stepwise approach—its universality—is already lost.

#### Contour Replacement Method

The target decomposition task implies a decomposition of the difference between states *A* and *B* conditioned on the past difference between *a* and *b* and the temporal changes from *a* to *A* and from *b* to *B*.

**Fig. 2:**
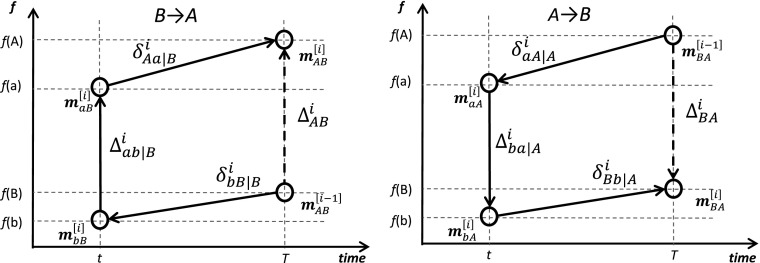
The *i*th step of the contour decomposition through transformation of vector **m**_*B*_ into vector **m**_*A*_ (left panel) and transformation of vector **m**_*A*_ into vector **m**_*B*_ (right panel)

Figure 2 presents the contour replacement graphically. Within each elementary age group *i*, the procedure includes a sequence of replacements over the contours *B→b→a→A* (left panel) and *A→a→b→B* (right panel).

Let us consider the former (clockwise) direction of replacement, which transforms vector **m**_*B*_ into vector **m**_*A*_ *.* The replacement sequence starts from the youngest age (*i* = 1). Following Eq. (6), the youngest age component of the cross-sectional difference at the second time point *T* is

13 $$ {\Delta}_{AB}^1=f\left({\mathbf{m}}_{AB}^{\left[1\right]}\right)-f\left({\mathbf{m}}_{\boldsymbol{B}}\right)=f\left({\mathbf{m}}_{AB}^{\left[1\right]}\right)-f\left({\mathbf{m}}_{AB}^{\left[0\right]}\right). $$

Using a simple algebraic trick, it is possible to express the component ${\Delta}_{AB}^1$ differently:

14 $$ {\Delta}_{AB}^1=\left[f\left({\mathbf{m}}_{bB}^{\left[1\right]}\right)-f\left({\mathbf{m}}_B\right)\right]+\left[f\left({\mathbf{m}}_{aB}^{\left[1\right]}\right)-f\left({\mathbf{m}}_{bB}^{\left[1\right]}\right)\right]+\left[f\left({\mathbf{m}}_{AB}^{\left[1\right]}\right)-f\left({\mathbf{m}}_{aB}^{\left[1\right]}\right)\right]. $$

The second step begins from the vector **B** with its first element having already been replaced by the first element of vector **A** *.* For the *i*th step, Eq. (6) can be written as follows:

15 $$ {\Delta}_{AB}^i=\left[f\left({\mathbf{m}}_{A(b)B}^{\left[i\right]}\right)-f\left({\mathbf{m}}_{AB}^{\left[i-1\right]}\right)\right]+\left[f\left({\mathbf{m}}_{A(a)B}^{\left[i\right]}\right)-f\left({\mathbf{m}}_{A(b)B}^{\left[i\right]}\right)\right]+\left[f\left({\mathbf{m}}_{AB}^{\left[i\right]}\right)-f\left({\mathbf{m}}_{A(a)B}^{\left[i\right]}\right)\right],i=1,\dots, n, $$

where

16 $$ {\mathbf{m}}_{A(a)B}^{\left[i\right]}=\left[{m}_A\left({x}_1\right),\dots, {m}_A\left({x}_{i-1}\right),{m}_a\left({x}_i\right),{m}_B\left({x}_{i+1}\right),\dots, {m}_B\left({x}_n\right)\right],\ {\mathbf{m}}_{A(a)B}^{\left[0\right]}={\mathbf{m}}_B. $$

**Fig. 3:**
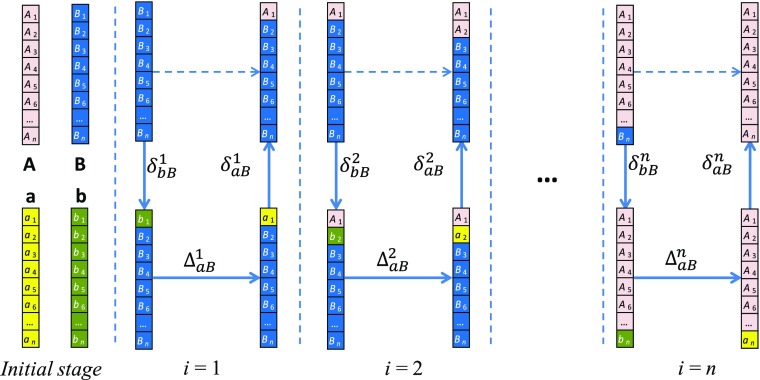
The sequence of element replacement in the four vectors in the direction *B→b→a→A*

The first additive term in Eq. (15) is the impact (on the function *f*) of the difference between *m*_*b*_(*x*_*i*_) and *m*_*B*_(*x*_*i*_), the second term represents the effect of the difference between *m*_*a*_(*x*_*i*_) and *m*_*b*_(*x*_*i*_), and the third component is the effect of the difference between *m*_*A*_(*x*_*i*_) and *m*_*a*_(*x*_*i*_). This equation corresponds to the replacement sequence depicted in the left panel of Fig. 2. Figure 3 further depicts the sequence of replacements at the level of each vectors’ elements. Instead of the direct replacement of the first element of vector **B** by the first element of vector **A** as is done in the conventional stepwise replacement algorithm (dashed arrow in Fig. 3), we pass through vectors **a** and **b** (solid arrows). At the second step, the replacement sequence is repeated for the second elements of all vectors and so on.

In Eq. (15), the first and the third additive terms are the trend (within-country) components of the change. They are produced by mortality changes in populations *B* (former *b*) and *A* (former *a*) and are defined as

17 $$ {\updelta}_{bB\mid B}^i=f\left({\mathbf{m}}_{A(b)B}^{\left[i\right]}\right)-f\left({\mathbf{m}}_{AB}^{\left[i-1\right]}\right),i=1,\dots, n $$

18 $$ {\updelta}_{Aa\mid B}^i=f\left({\mathbf{m}}_{AB}^{\left[i\right]}\right)-f\left({\mathbf{m}}_{A(a)B}^{\left[i\right]}\right),i=1,\dots, n. $$

The second additive term in Eq. (15) is an initial conditions (between-country) component of the change

19 $$ {\Delta}_{ab\mid B}^i=f\left({\mathbf{m}}_{A(a)B}^{\left[i\right]}\right)-f\left({\mathbf{m}}_{A(b)B}^{\left[i\right]}\right),i=1,\dots, n. $$

An alternative (counterclockwise) replacement path *A→a→b→B* can be expressed by

20 $$ {\Delta}_{BA}^i=\left[f\left({\mathbf{m}}_{B(a)A}^{\left[i\right]}\right)-f\left({\mathbf{m}}_{BA}^{\left[i-1\right]}\right)\right]+\left[f\left({\mathbf{m}}_{B(b)A}^{\left[i\right]}\right)-f\left({\mathbf{m}}_{B(a)A}^{\left[i\right]}\right)\right]+\left[f\left({\mathbf{m}}_{BA}^{\left[i\right]}\right)-f\left({\mathbf{m}}_{B(b)A}^{\left[i\right]}\right)\right],i=1,2,\dots n. $$

The corresponding trend and initial conditions’ components of the change are

21 $$ {\updelta}_{aA\mid A}^i=f\left({\mathbf{m}}_{B(a)A}^{\left[i\right]}\right)-f\left({\mathbf{m}}_{BA}^{\left[i-1\right]}\right),i=1,\dots, n $$

22 $$ {\updelta}_{Bb\mid A}^i=f\left({\mathbf{m}}_{BA}^{\left[i\right]}\right)-f\left({\mathbf{m}}_{B(b)A}^{\left[i\right]}\right),i=1,\dots, n $$

23 $$ {\Delta}_{ba\mid A}^i=f\left({\mathbf{m}}_{B(b)A}^{\left[i\right]}\right)-f\left({\mathbf{m}}_{B(a)A}^{\left[i\right]}\right),i=1,\dots, n. $$

The final trend components are determined by averaging the two equally possible contour paths corresponding to Eqs. (17)–(19) and (21)–(23):

24 $$ {\updelta}_{Aa\mid AB}^i=\frac{1}{2}\ \left[{\updelta}_{Aa\mid B}^i-{\updelta}_{aA\mid A}^i\right] $$

25 $$ {\updelta}_{Bb\mid AB}^i=\frac{1}{2}\ \left[{\updelta_{Bb\mid B}^i-\updelta}_{bB\mid A}^i\right]. $$

Equations (24) and (25) are components contributed by trends in each of the two populations being compared. The total trend component produced by the interpopulation difference in trends is

26 $$ {Trend}^i={\updelta}_{ab\mid AB}^i={\updelta}_{Aa\mid AB}^i-{\updelta}_{Bb\mid AB}^i. $$

Similarly, the initial conditions component is

27 $$ {Initial}^i={\Delta}_{ab\mid AB}^i=\frac{1}{2}\ \left[{\Delta}_{ab\mid B}^i-{\Delta}_{ba\mid A}^i\right]. $$

Equations (17)–(19) and (21)–(27) fully determine the algorithm of contour replacement. Equations (15) and (20) ensure that the condition specified by Eq. (4) holds true for every age *x*_*i*_:

$$ {\Delta}_{AB}^i={Initial}^i+{Trend}^i={\Delta}_{ab\mid AB}^i+{\updelta}_{ab\mid AB}^i,\kern0.75em i=1,2,\dots, n. $$

### Comparison of the Additive Change and Contour Replacement Methods

The results of the additive change method and the contour replacement method are likely to be similar when the former method is applicable: that is, *f*(**m** + Δ**m**) is defined for any ${\mathbf{m}}_{ab}^{\left[i-1\right]}+\Delta {\mathbf{m}}_{AB}^{\left[i\right]},{\mathbf{m}}_{ab}^{\left[i\right]}+\Delta {\mathbf{m}}_{AB}^{\left[i\right]}$ and ${\mathbf{m}}_{ab}^{\left[i\right]}+\Delta {\mathbf{m}}_{AB}^{\left[i-1\right]},i=1,\dots, n$. It implies that all age-specific elements of these vectors are nonnegative. It is possible to show that returns of the two methods will be similar if, for small differences (*m*_*A*_(*x*_*i*_) − *m*_*a*_(*x*_*i*_)), the index function and values of independent variables are such that the following linearization holds true.

28 $$ f\left({\mathbf{m}}_{A(a)A}^{\left[i\right]}\right)-f\left({\mathbf{m}}_A\right)\approx \frac{\partial f}{\partial {m}_A\left({x}_i\right)}\left({m}_A\left({x}_i\right)-{m}_a\left({x}_i\right)\right). $$

(For more details, see Online Resource 3.) Equation (28) implies that the two methods return identical results for linear functions and are very close for many functions that experience small changes in response to small changes in independent covariates (i.e., age-specific death rates). More substantial disagreement may be expected for measures that experience large changes in response to small changes in covariates. Most life table–based indices have moderate sensitivity corresponding to minor differences between the left and the right sides of Eq. (28).

To empirically verify that the contour and the additive change decomposition methods produce similar results for life table applications, we compared the two approaches on several aggregate life table indices using life table data from the Human Mortality Database (HMD) (n.d.). Pairwise comparisons were done between all countries 10, 20, and 30 years apart on the first year of each decade for each sex, using single year of age and single calendar year (1 × 1), and five-year age categories and single calendar year (5 × 1) life tables. Countries with small populations were excluded, as were some country-year combinations that were missing data around the time of the world wars. This left us with 6,646 decompositions 10 years apart; 5,456 decompositions 20 years apart; and 4,266 decompositions 30 years apart. The full list of life tables used is given in Online Resource 4.

Recall from the previous section that the additive change decomposition method was akin to decomposition by age and cause of death, where the initial vector of death rates (**m**) and the vector of the change in death rates (Δ**m**) were considered as analogs of two vectors of cause-specific death rates. However, unlike replacement between nonnegative death rates for causes of death, in this new formulation, replacement of **m** and Δ**m** between-populations can result in cases where the change in mortality is greater than the initial mortality. An instance of this can be found in the empirical example comparison of the United States and England and Wales (1980–2010) made in the section that follows. When we stepwise replaced the female vectors **m** and Δ**m**, the mortality reduction in the United States at age 15 (–0.00021) was greater than the initial mortality rate in England and Wales at age 15 (0.00020). The sum of these terms is negative, and it was thus not possible to determine the initial and trend components for this age using the additive change method.

Overall, this turned out to be a nontrivial problem. For the single year of age life tables compared 10 years apart, negative elements of the vector **m** + Δ**m** were produced in at least one age group for 65 % of the decompositions (Table 1). When the decomposition window was extended to 30 years, fully 95 % of the decompositions had at least one problematic age. Negative death rates were more likely to occur in low mortality settings, especially over young ages with few death counts. Although the use of abridged 5 × 1 life tables reduced the number of such problems, still one-quarter of decompositions between countries 10 years apart produced negative death rates over at least one age group.

**Table 1:** A summary of the applicability^a^ of the additive change method

		Negative *m*(*x*) Produced		
Age × Year Scale	*T* – *t* Interval	Number	Proportion	Number of Decompositions
1 × 1	10 years	4,293	.65	6,646
1 × 1	20 years	4,896	.90	5,456
1 × 1	30 years	4,044	.95	4,266
5 × 1	10 years	1,760	.26	6,646
5 × 1	20 years	2,719	.50	5,456
5 × 1	30 years	3,023	.71	4,266

^a^When the mortality change is larger than the initial difference in mortality, a negative *m*(*x*) is produced over the age range, which then does not permit the additive change method to be used.

We then contrasted the initial and the trend components produced by the contour and additive change decomposition methods. The two methods produced very similar results for life expectancy, lifetime disparity, the Theil index, and the average interindividual difference between ages at death in cases when the additive change method was applicable (see Online Resource 4).

## Empirical Example: Mortality Development of the United States and England and Wales

The United States ranks poorly among developed countries in life expectancy at birth.^3^ Moreover, as we acknowledged in the beginning of this article, the United States has substantially higher lifespan variation than other developed countries, including English-speaking countries. This is not strictly due to Americans having lower life expectancy: even at similar life expectancy levels or similar modal ages at death, the United States has comparatively higher lifespan variation (Edwards and Tuljapurkar 2005; Shkolnikov et al. 2003, 2011; Smits and Monden 2009; Vaupel et al. 2011; Wilmoth and Horiuchi 1999).

We return to the question posed at the beginning of this study: to what extent can current differences in life expectancy and lifetime disparity be explained by age-specific mortality trends since 1980 in the United States and in England and Wales? We complement this question by looking at the role which different age groups play in explaining divergence in life expectancy at age 40 for the 1900 and 1920 birth cohorts. The method proposed in this study is new and the only method (so far) that can correctly answer this question by comparing trends while controlling for initial differences in age-specific mortality levels.

Comparing the United States with England and Wales is particularly interesting. The two countries share strong historic, linguistic, and cultural ties, yet health and welfare policies are markedly different. England and Wales have a long tradition of publicly funded health care provided at all ages, whereas American health care coverage varies widely depending on age, employment status, and disability, costing more than twice as much per capita (OECD 2016). Americans report higher levels of disease, fare worse on a host of biomarkers than the English and Welsh, and have stronger socioeconomic health gradients at middle age (Banks et al. 2006).

Shkolnikov et al. (2011) compared the mortality development of the United States and England and Wales through a series of temporal and between-country decompositions. They found that the two countries experienced mortality reduction from 1980 to 2003 from similar causes of death but that in both the early and later periods, between-country differences were driven by a different set of causes of death, especially causes amenable to medical intervention, accidents, and violence. However, because the sum of the between-country and temporal decompositions do not sum to the current gap in life expectancy (*e*_0_) or lifetime disparity (*e* ^†^) (as explained in the Introduction, the Decomposition Task, and the Between-Population and Within-Population Decompositions sections), they did not have a method to quantify correctly the age-specific contributions of the past mortality differences and the differential mortality change to the Anglo-American differences in life expectancy or in lifetime disparity observed in the 2000s.

Using our new contour decomposition method, we decomposed recent gaps in life expectancy and lifetime disparity into the age-specific initial conditions component and age-specific trend contributions. In an update to the previous study by Shkolnikov et al. (2011), we considered the mortality change from 1980 to 2010. We used HMD life table data and our R code, which is freely available (Jdanov and Shkolnikov 2014) so that the following example can be easily replicated.

To recap from the method description, to apply the decomposition method, we designated the American populations as *A* (2010) and *a* (1980). England and Wales were designated as *B* (2010) and *b* (1980). All life expectancy and lifetime disparity differences and age-specific contributions to those differences in the text, tables, and figures that follow are computed as “United States minus England and Wales.” The contour decomposition method began by replacing the age-specific death rates, *m*(*x*), along the age-period contour starting from the youngest age: that is, *m*_*A*_(*x*_0_) → *m*_*a*_(*x*_0_) → *m*_*b*_(*x*_0_) → *m*_*B*_(*x*_0_); *m*_*A*_(*x*_1_) → *m*_*a*_(*x*_1_) → *m*_*b*_(*x*_1_) → *m*_*B*_(*x*_1_); . . . ; *m*_*A*_(*x*_110+_) → *m*_*a*_(*x*_110+_) → *m*_*b*_(*x*_110+_) → *m*_*B*_(*x*_110+_). After each replacement step, life expectancy and lifetime disparity were recalculated. The difference in the new *e*_0_ and *e* ^†^ from the previous replacement’s *e*_0_ and *e* ^†^ produced three contributions at each age: a U.S. trend *A* →*a* contribution, an initial between-population contribution *a* → *b*, and an England and Wales trend contribution *b* → *B*. The overall trend contribution is the sum of the U.S. and the England and Wales trends. Afterward, we performed all steps in reverse—that is, *m*_*B*_(*x*_0_) → *m*_*b*_(*x*_0_) → *m*_*a*_(*x*_0_) → *m*_*A*_(*x*_0_)—until we finished with replacements *m*_*B*_(*x*_110+_) → *m*_*b*_(*x*_110+_) → *m*_*a*_(*x*_110+_) → *m*_*A*_(*x*_110+_). The initial and trend contributions were averaged over the two replacement directions. Finally, we performed a traditional stepwise decomposition of the final Anglo-American difference *A* → *B* and *B* → *A* as a control to show that the initial and trend age-specific components summed to the final age-specific decomposition components.

Between 1980 and 2010, the United States and England and Wales experienced diverging life expectancy for both sexes but smaller change in the lifetime disparity gap (Table 2). Life expectancy increased substantially more for men than for women, especially in the United States. In 1980, American women had a 0.7-year advantage (+0.9 %) in life expectancy over English and Welsh women; by 2010, they had a disadvantage of 1.3 years (–1.6 %). Over the same period, the life expectancy disadvantage of American men increased from 0.7 years (–1 %) to 2.3 years (–3 %).

**Table 2:** Differences in life expectancy and lifetime disparity between the United States and England and Wales, 1980–2010: Data from the human mortality database

	Life Expectancy (*e*_0_)			Lifetime Disparity (*e* ^†^)		
	United States	England and Wales	Difference	United States	England and Wales	Difference
Men						
1980	70.0	70.7	–0.7	13.4	11.6	1.8
2010	76.4	78.6	–2.3	12.2	10.6	1.6
Increase	6.4	7.9	–1.5	–1.2	–1.0	–0.2
Women						
1980	77.5	76.8	0.7	12.0	11.0	1.0
2010	81.2	82.6	–1.4	10.9	9.7	1.1
Increase	3.7	5.8	–2.1	–1.1	–1.3	0.2

For lifespan variation, American women had lifetime disparity levels that were 1.0 (1980) and 1.2 (2010) years higher than women in England and Wales (+8.7 % and +11.7 %, respectively), but the surplus life disparity of U.S. men decreased from 1.8 to 1.6 years (+14.4 % and +14 %, respectively).

**Fig. 4:**
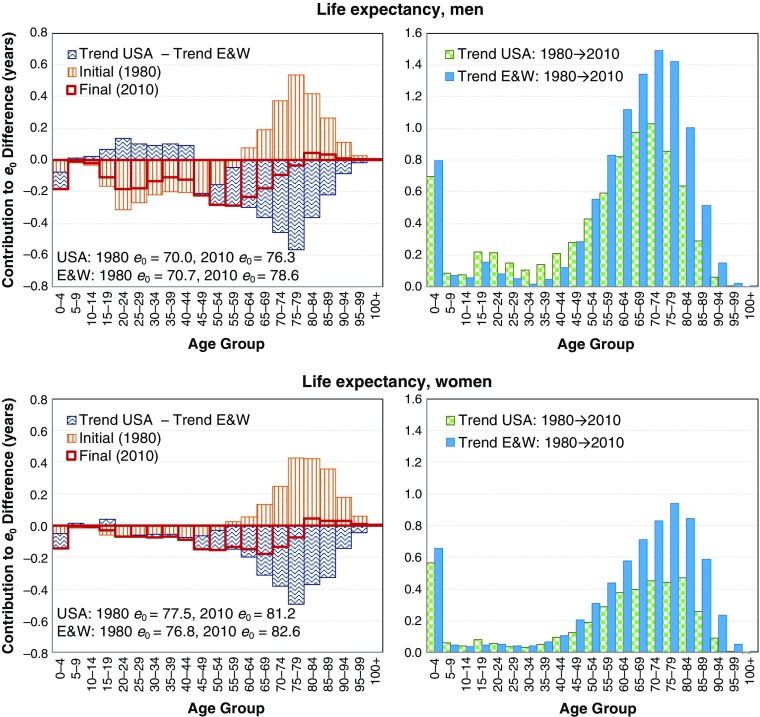
Contour decomposition of the 2.3-year (men) and 1.4-year (women) life expectancy gap between the United States and England and Wales in 2010, looking back to the development since 1980 (left panel) and separated trend components of the United States and England and Wales in contour decomposition (right panel)

The contour decomposition results for life expectancy are displayed in Fig. 4. For men, the open bars with solid border (upper-left panel) show the age-specific contributions to the 2010 life expectancy gap of 2.3 years between the United States and England and Wales—results that are obtained from traditional between-population decomposition. Infancy, early adult mortality and midlife mortality were the main contributors to this gap. After age 80, the United States retained a small advantage of about one-tenth of a year in life expectancy (see Table 3).

**Table 3:** The contour decomposition of the United States and England and Wales 2010 period life expectancy and lifespan disparity difference, looking back to the initial 1980 period

	Women			Men		
Age	Initial Component	Trend Component	Conventional Decomposition in 2010	Initial Component	Trend Component	ConventionalDecomposition in 2010
Life Expectancy						
0–19	–0.14	–0.04	–0.18	–0.31	–0.01	–0.32
20–39	–0.24	–0.04	–0.28	–1.02	0.42	–0.61
40–59	–0.15	–0.37	–0.52	–0.64	–0.28	–0.92
60–79	0.86	–1.40	–0.54	1.16	–1.71	–0.54
80+	0.94	–0.82	0.12	0.71	–0.62	0.09
Total	1.26	–2.67	–1.41	–0.10	–2.21	–2.30
Lifetime Disparity						
0–19	0.12	0.04	0.16	0.25	0.01	0.27
20–39	0.20	0.03	0.23	0.79	–0.32	0.47
40–59	0.11	0.25	0.36	0.41	0.15	0.56
60–79	–0.23	0.47	0.23	–0.08	0.27	0.19
80+	0.70	–0.60	0.10	0.73	–0.64	0.09
Total	0.90	0.19	1.09	2.11	–0.53	1.58

*Notes:* The initial component reflects age-specific contributions relating to the 1980 mortality difference; the trend component accounts for the effect of different age-specific mortality trends from 1980 to 2010. The conventional decomposition column presents both the age-specific results of a traditional stepwise decomposition of the 2010 difference and the sum of the initial and trend components.

These age-specific contributions were then decomposed on components owing to initial 1980 differences in mortality and contributions from age-specific mortality trends 1980–2010. The trend components are the differences between contributions of changes in mortality in the United States and respective contributions of changes in mortality in England and Wales (right panel of Fig. 4). Below age 60, American men already had higher mortality rates than in England and Wales in 1980, illustrated by the initial contributions line. However, over the 30-year period, Americans reduced the mortality gap over some of these ages. In particular, the comparatively faster mortality decline in the United States over ages 1–45 lowered the life expectancy gap by 0.6 years. Yet, even with the comparatively stronger mortality decline, these ages still contribute 0.9 years to the American shortfall because of the much higher initial rates in the United States. Between ages 45 and approximately 60, American men had both higher initial mortality levels and experienced slower mortality decline. As a result, the contribution of these ages to the life expectancy gap increased. After age 60, American men had lower initial mortality than the English and Welsh but experienced slower mortality decline. The crossover age in mortality pushed upward from around 60 to 80, and even above age 80, the American mortality advantage was severely weakened. Thus, after controlling for different initial mortality rates, we can say that weaker U.S. trends in infancy and above age 45 contributed 0.1 and 2.8 years, respectively, to the life expectancy shortfall of the United States. Stronger mortality decline between ages 1 and 45 reduced this shortfall by 0.6 years. Altogether, this summed to the 2.3-year male life expectancy gap between the two countries in 2010.

For women, the age pattern of mortality difference did not change as much over the period. Women in England and Wales also had lower mortality below age 50, but the difference between countries was much smaller than that among the men, and female trends in the two countries over the period were similar. However, the United States held a life expectancy advantage in 1980 owing to lower mortality above age 50. Over the period 1980–2010, England and Wales experienced stronger mortality decline over all ages, and the life expectancy advantage of the United States changed to more than a one-year disadvantage. The largest contributions to this change were produced by ages around 80.

The initial age and final age patterns of mortality difference, shown in Fig. 4, explain why England and Wales had lower lifetime disparity than the United States in both periods. The lower mortality in England and Wales over younger ages compresses mortality into a narrower age range, while higher mortality over older ages ensures a heavier right tail of the age at death distribution. In between lies a threshold age separating the effect of mortality decline from these younger compressing ages and older expanding ages (for additional insights, see also Gillespie et al. 2014; van Raalte and Caswell 2013; Zhang and Vaupel 2009).

**Fig. 5:**
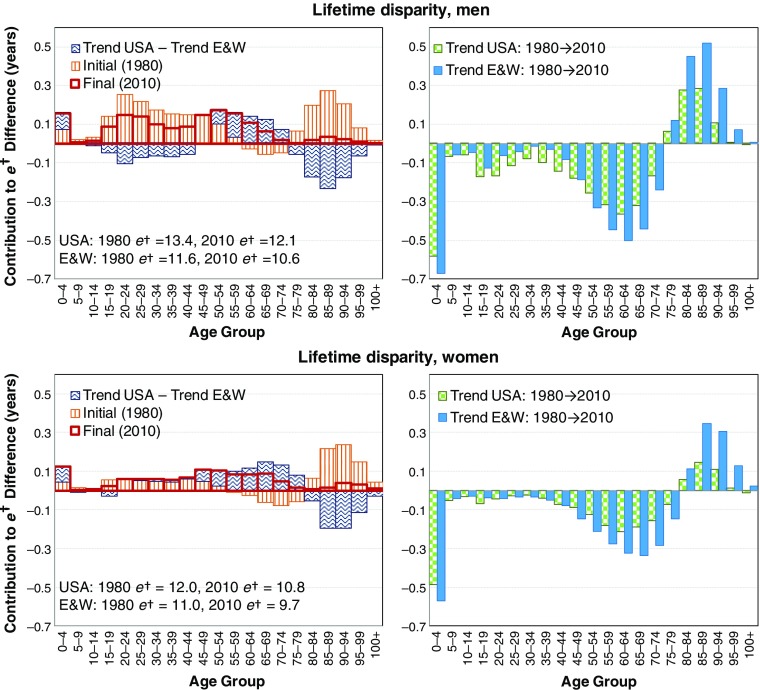
Contour decomposition of the 1.6-year (men) and 1.1-year (women) higher lifetime disparity experienced by the United States over England and Wales in 2010, looking back over the development since 1980 (left panel) and separated trend components of the United States and England and Wales in contour decomposition (right panel)

In other words, stronger trends in mortality decline at any age for the United States would narrow the U.S. life expectancy disadvantage. To lower the lifetime disparity gap (i.e., reduce the high disparity in the United States compared with England and Wales), however, mortality decline would have to be either comparatively stronger over younger ages or weaker over older ages. The contour decomposition results for lifetime disparity are shown in Fig. 5. Among men, the comparatively stronger U.S. mortality decline over ages 1–45 reduced the lifetime disparity gap; over ages 45–75, the weaker mortality decline increased the gap; but above age 75, the weaker mortality decline decreased the lifetime disparity gap. For women, similar mortality decline between the two countries below age 50 left the gap unchanged, weaker U.S. decline from ages 50 to 80 increased the gap, and weaker U.S. decline from ages above age 80 decreased the gap. The net effect of these age-specific changes for both sexes was that the lifetime disparity gap remained mostly unchanged. Nevertheless, the shift to a later crossover age in mortality advantage for the United States meant that by 2010, almost all ages were contributing to larger lifetime disparity in the United States.

Thus, Figs. 4 and 5 suggest that much of the mortality change over the period was actually a convergence in age patterns of mortality between the two countries. For men, the United States was catching up on mortality decline over young ages where it had a large survival disadvantage, and England and Wales was catching up on the U.S. old-age advantage. For women, differences over younger ages were not so large, and the trends were mostly a case of England and Wales narrowing and in some cases (women) overtaking the survival gap with the United States at older ages.

As a second example, we turn to cohort change. Currently, it is unclear whether the recent temporal change in mortality decline is due to period or cohort processes (Murphy 2010). Nevertheless, the contour decomposition method can equally be applied to cohorts. American time series of mortality from the HMD begin as late as 1933. Thus, we do not have complete information for any cohort; however, we have enough data to compare changes in remaining life expectancy at age 40 for the 1900 and 1920 cohorts in the two countries.

**Fig. 6:**
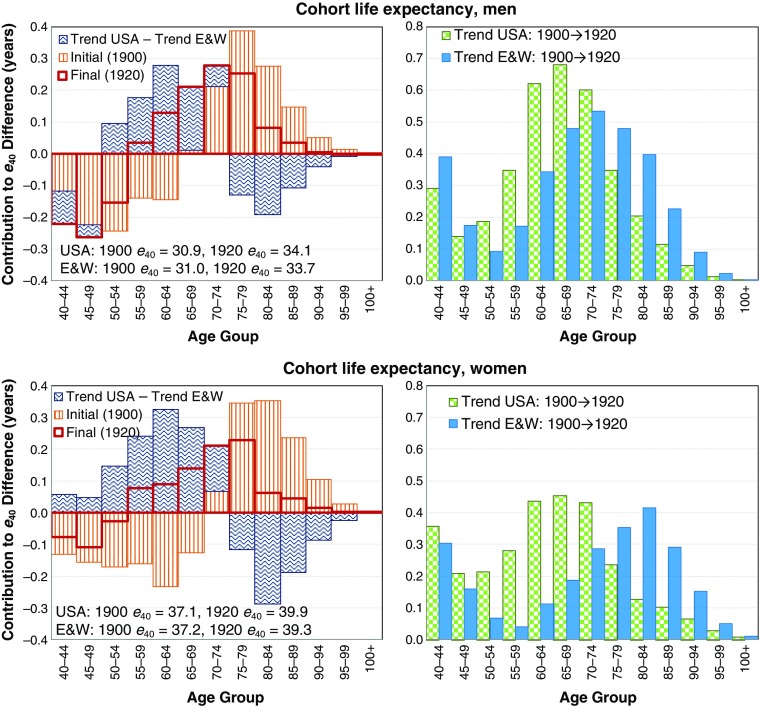
Contour decomposition of the 0.4-year (men) and 0.6-year (women) cohort life expectancy at age 40 advantage of the United States over England and Wales for the 1920 cohorts, looking back to the development since the 1900 cohorts (left panel) and separated trend components of the United States and England and Wales in contour decomposition (right panel)

In Fig. 6, we show the results of such a contour decomposition. As observed in the period figures, later cohorts in the two populations are converging to more similar age patterns of mortality (the magnitude of open bars is lower), but differences are less striking than in the period example. The 1900 U.S. cohorts had higher midlife mortality and lower old age mortality than the 1900 England and Wales cohorts. As in the period example, the United States experienced weaker mortality declines over older ages. However, unlike in the period example, the United States experienced comparatively stronger mortality decline than England and Wales over middle adult ages (49–73 for men, and 41–74 for women) from the 1900 to 1920 cohorts. Overall, the especially weak female trends are not evident for these cohorts: mortality change turned a cohort life expectancy deficit to an advantage for the United States over England and Wales. Note that the 1900 birth cohort would have been age 80 in 1980 and age 110 in 2010, while the 1920 birth cohorts would have been ages 60 and 90, respectively. Thus, the individuals displayed in the cohort example would all be in the older age categories in the period example. However, it would also be possible to compare contour decomposition results for younger cohorts in temporary (interval) life expectancy up to the highest age observed in order to capture the American cohorts that have experienced particularly weak trends in recent years.

## Discussion

### Added Value

We began this article with a comparison between the United States and England and Wales. At present, the United States experiences lower life expectancy and much higher lifetime disparity than England and Wales. The conventional decompositions of these differences would show that mortality at nearly all ages contribute to these differences with larger components pertaining to older working ages and smaller components pertaining to younger and older ages. The newly developed contour decomposition method allows better measurement and understanding of the origins of this pattern. We could see, for example, that the high American mortality among young adult men is still an important contributor to the intercountry gap in both life expectancy and lifetime disparity but that this feature is mostly a legacy of the past, which has diminished by approximately one-third because of mortality progress among young adults in the United States. We could also see that at older ages, the current moderate values of age components result from two counterbalancing forces: the substantial initial mortality advantage of the United States in old-age mortality and mortality trends that brought the English and Welsh mortality at old ages much closer to the low but slowly declining mortality of the American elderly.

This finding suggests that the age pattern behind the exceptionally high lifetime disparity in the United States is slowly changing. The former outlying pattern that combines high mortality among young adults with low mortality among the elderly is becoming weaker. Slower American mortality declines at older working ages and old ages are becoming more important contributors to the gap in life expectancy and lifetime disparity between the United States and other countries. To a large extent, this finding on comparatively strong early adult mortality decline challenges the recent highly publicized account of poor trends in American mortality over these ages (Case and Deaton 2015), partially because we are examining the entire American population by sex, whereas Case and Deaton limited their analysis to white non-Hispanic Americans of both sexes. Gains by other race groups have been comparatively strong during these 30 years (Harper et al. 2007, 2012). We also found that the young adult mortality convergence was limited to men. Women continued to lose ground in life expectancy over most adult ages, including these ages, although in absolute terms poor trends over ages 75–85 were contributing the most to widening female life expectancy differences.

Finally, we limited our comparison to England and Wales, which experience more similar losses in life expectancy to the United States from behavior-driven mortality than other Western European countries (Preston and Stokes 2011; Preston et al. 2010). Future work should situate patterns of mortality change in a broader comparative setting.

The Anglo-American comparison illustrates how the decomposition method developed in this study enables the investigation of the origins of observed intercountry differences by quantifying the relative importance of initial mortality conditions and trends in these differences. To date, a scholar would normally have approached this question by separately examining the components of between-population differences in the past and components of mortality change between the starting and present periods. Information obtained from such analysis would include differences and changes attributed to each elementary event rate. These differences and changes are, however, incomparable, and their age-specific totals do not equal the respective age-specific components of the final intercountry difference, which may lead to conflicting evidence. In contrast, the decomposition method proposed in this study permits a difference in an aggregate measure at a final time point to be split into additive components that correspond to the initial differences in the event rates of the measure and differences in trends in these underlying event rates.

In this study, the aggregate measure was defined as a function of a vector of age-specific death rates. Technically, our target decomposition task was to develop a decomposition method that ensured that the sum of the initial conditions and trend components equaled the conventional age component (i.e., from other decomposition methods) of the between-population difference at the final time point. We accomplished this by using the contour replacement and additive change algorithms. We advocate in favor of the contour replacement method because of its universal applicability to mortality change given any combination of life tables. Moreover, we used the stepwise replacement algorithm as the baseline method, but the idea of changes moving along a demographic contour is easily transferable to any known decomposition method, while the implementation of an additive change approach would be more complicated. However, we caution that errors accumulate the wider the time window being examined.

Both the contour replacement and additive change decomposition methods were built on the general stepwise decomposition approach. Perhaps another method based on a continuous change model (Caswell 2001; Horiuchi et al. 2008) could be developed for solving the same problem. Similar to what is found for conventional between-population decompositions, different approaches could produce slightly different numerical results.

In the present study, we considered the aggregate measure to be a function of a one-dimensional vector. It is certainly possible to include additional dimensions of interest in the decomposition, such as causes of death. In this case, each step of the contour replacement of a single age-specific death rate would include a sequence of replacements of age- and cause-specific rates. This is analogous to the inclusion of additional dimensions within the framework of the general stepwise replacement algorithm as described in earlier studies (Andreev et al. 2002; Shkolnikov et al. 2011).

### Limitations

The use of our new decomposition technique requires predefining the initial and final time points. The initial and trend components will obviously differ, depending on the period under consideration. In addition, because of the path dependence of mortality change, the sum over a sequence of yearly decompositions—that is, 1990 → 1991, 1991→1992, . . . , 2009 → 2010—will produce somewhat different initial and trend components compared with decomposing directly over the period 1990→2010. However, this limitation is inherent to the defined objective of the study.

As with the original stepwise replacement method, decomposition was undertaken in a logical ordering of young to old age. This is consistent with other conventional decompositions by formula (Andreev 1982; Arriaga 1984; Pressat 1985), although Pollard (1988) used an average of old-to-young and young-to-old orderings. The young-to-old ordering used here produced age components similar to those found by Horiuchi et al. (2008) using a continuous decomposition method. Clearly, other or random age replacement paths are possible as well. With three ages, there are six potential pathways of replacement. With 10 ages, there are 10 factorial (3,628,800) possible pathways, already a number that is computationally intensive if not infeasible. Simulations of completely random age-specific mortality replacement ordering have shown the differences in the estimated age components of a stepwise life expectancy decomposition to be minor (Andreev et al. 2002).

### Conclusion

The new decomposition method proposed by this study allows quantification of the effect of past mortality conditions and temporal change within each age component of a contemporary difference in aggregate demographic indices. In this study, we demonstrated that the method is applicable to a range of life table indices. It can be applied to other aggregate indices describing not only mortality but also fertility, population reproduction, and migration. We hope that together with the freely available R script, the contour replacement method has a chance to become a useful instrument of the general demographic toolkit.

### Electronic supplementary material

Online Resource 1 (PDF 387 kb)

Online Resource 2 (PDF 408 kb)

Online Resource 3 (PDF 408 kb)

Online Resource 4 (PDF 517 kb)

## Appendix

**Table 4:** Mathematical abbreviations used in the study

Notation	Description
*T*	Final time point
*t*	Initial time point
*A,B*	Populations at final time point *T*
*a,b*	Populations at initial time point *t*
*E* = *f*(.)	Demographic measure (index) to be decomposed
**m**_*A*_	Vector of age-specific event rates [*m*_*A*_(*x*_1_), . . . ,*m*_*A*_(*x*_*i*_), . . . ,*m*_*A*_(*x*_*n*_)]
*m*_*A*_(*x*_*i*_)	Age-specific event rate for age interval [*x*_*i*_ *, x*_*i+*1_)
Δ_*AB*_	Between-population difference in index *E*
${\Delta}_{AB}^i$	Age-specific components of between-population difference in index *E*
δ_*Aa*_	Within-population difference in index *E* due to temporal mortality changes
${\updelta}_{Aa}^i$	Age-specific components of within-population difference in index *E*
${\mathbf{m}}_{AB}^{\left[i\right]}$	[*m*_*A*_(*x*_1_), . . . ,*m*_*A*_(*x*_*i*_), *m*_*B*_(*x*_*i* + 1_), . . . ,*m*_*B*_(*x*_*n*_)]
Δ**m**_*A*_	**m**_*A*_ − **m**_*a*_
${\Delta \mathbf{m}}_{AB}^{\left[i\right]}$	[∆*m*_*A*_(*x*_1_), . . . ,∆*m*_*A*_(*x*_*i*_), ∆*m*_*B*_(*x*_*i* + 1_), . . . ,∆*m*_*B*_(*x*_*n*_)]
${\Delta}_{+ ab\mid AB}^i$	Age-specific (age *i*) initial component of the additive change method
${\Delta}_{+ ab}^i$	Age-specific initial component of the additive change method by the replacement path *B→b→a→A*
${\updelta}_{+ ab\mid AB}^i$	Age-specific trend component of the additive change method
${\updelta}_{+ ab}^i$	Age-specific trend component of the additive change method by the replacement path *B→b→a→A*
${\mathbf{m}}_{A(a)B}^{\left[i\right]}$	$\left[{m}_A\left({x}_1\right),\dots, {m}_A\left({x}_{i-1}\right),{m}_a\left({x}_i\right),{m}_B\left({x}_{i+1}\right),\dots, {m}_B\left({x}_n\right)\right],\kern0.75em {\mathbf{m}}_{A(a)B}^{\left[0\right]}={\mathbf{m}}_B$
${\updelta}_{ab\mid AB}^i$	Age-specific trend component by contour decomposition
${\updelta}_{ab\mid B}^i$	Age-specific trend component of contour decomposition by replacement path *B→b→a→A*
${\Delta}_{ab\mid AB}^i$	Age-specific initial component of contour decomposition
${\Delta}_{ab\mid B}^i$	Age-specific initial component of contour decomposition by replacement path *B→b→a→A*

## Footnotes

1 For simplicity, the elementary age interval [*x*_*i*_ *, x*_*i+*1_) is designated by its beginning age *x*_*i*_ in this and later formulae.

2 Note that generally speaking,

$$ \begin{array}{l}{\Delta}_{ab}^i\ne {Initial}^i,{\Delta}_{ab}\ne {\sum}_{i=1}^n{Initial}^i\hfill \\ {}{\updelta}_{Aa}^i-{\updelta}_{Bb}^i\ne {\updelta}_{ab\mid AB}^i,{\updelta}_{Aa}-{\updelta}_{Bb}\ \ne\ {\sum}_{i=1}^n{\updelta}_{ab\mid AB}^i.\hfill \end{array} $$

That is, as we mention in the Introduction, the conventional components of the initial difference between *a* and *b* are not equivalent to the contributions of this initial difference to the final difference between *A* and *B* (denoted as ${\Delta}_{ab\mid AB}^i$). Similarly, the differences between conventional components of the within-population temporal changes are not equivalent to the contributions of the temporal change to the final difference between *A* and *B* (denoted as ${\updelta}_{ab\mid AB}^i$). This is further elaborated in the next section.

3 Recently, the National Research Council–appointed Panel on Understanding Divergent Trends in Longevity in High-Income Countries was commissioned to investigate why the United States has been falling behind in life expectancy rankings of developed countries at ages 50 and above (Crimmins et al. 2011). Among the main findings were that males have consistently had among the lowest *e*_50_ rankings over the last half of the twentieth century, while females experienced less than half the gains in *e*_50_ compared with the top performers over the 1980–2004 period. A key question in the report was the extent to which the current U.S. lifespan disadvantage could be attributed to recent age-specific mortality trends (Glei et al. 2010). Although the authors compared the contributions of different age groups with life expectancy gain in the United States versus gain in a wealthy country composite, it was unclear whether trends were weaker in some age groups because of slow progress or because initial levels were already low over these ages.
