# Supplementary material for: Decomposing Current Mortality Differences Into Initial Differences and Differences in Trends: The Contour Decomposition Method
Source: Demography. 2017 Jul 28;54(4):1579–602. doi: 10.1007/s13524-017-0599-6 (PMC5547192; doi:10.1007/s13524-017-0599-6)
Supplement: Supplementary file 1 — (PDF 387 kb) [file 13524_2017_599_MOESM1_ESM.pdf]

Online Resource 1. Summary of the stepwise replacement algorithm

The idea of the stepwise replacement algorithm is to present decomposition of the total change in an index function *f*(.) as a sequence of replacement of its arguments. Let *f*(.) depend on (say) three covariates *u*, *v*, and *w* which are defined at time points 1 and 2. The difference

$$\Delta f = f(u_2, v_2, w_2) - f(u_1, v_1, w_1)$$

can be presented as a sequence of replacements:

$$\Delta f = \Delta f(u_2 \to u_1, v_1, w_1) + \Delta f(u_2, v_2 \to v_1, w) + \Delta f(u_2, v_2, w_2 \to w_1)$$

with the *delta* components

$$\Delta f(u_2 \to u_1, v_1, w_1) = f(u_2, v_1, w_1) - f(u_1, v_1, w_1);$$

$$\Delta f(u_2, v_2 \to v_1, w) = f(u_2, v_2, w_1) - f(u_2, v_1, w_1);$$

$$\Delta f(u_2, v_2, w_2 \to w_1) = f(u_2, v_2, w_2) - f(u_2, v_2, w_1).$$

Each of these three components corresponds to the contribution of the shift in value of the respective covariate from point 1 to point 2. Note that once the value of the independent variable (say *u*) is shifted from u_1 to u_2, it remains equal to u_2 when the delta-components of the covariates that come later in the replacement sequence are calculated.

The three components given above correspond to the replacement sequence *u*-*v*-*w*. It is possible, however, to move from $u_1v_1w_1$ to $u_2v_2w_2$ using other pathways. For example, it is possible to carry out the replacement sequence *v*-*u*-*w* ($v_2 \to v_1$, $u_2 \to u_1$, $w_2 \to w_1$). The number of all possible replacement sequences equals 6 (all possible permutations among three elements). For a non-linear function $f(u, v, w)$, values of the component produced by a movement from point 1 to point 2 for the same covariate in different replacement sequences are not exactly equal to one another. For example, the *u*-component

$$\Delta \mathrm{f}(u_2 \to u_1, v_2, w_1) = f(u_2, v_2, w_1) - f(u_1, v_2, w_1)$$

in sequence *v*-*u*-*w* is not exactly the same as the *u*-component in sequence *u*-*v*-*w*

$$\Delta f(u_2 \to u_1, v_1, w_1) = f(u_2, v_1, w_1) - f(u_1, v_1, w_1).$$

Thus, the stepwise replacement has to be carried out for all possible replacement sequences (permutations) and the final components are to be computed as averages of the components' values over all these sequences (Andreev et al. 2002; Das Gupta 1994; Horiuchi et al. 2008). In our example it means that the component corresponding to each covariate (*u*, *v*, or *w*) should be calculated as the average of six respective sequence-specific component values.

The stepwise replacement algorithm with the full run across all replacement sequences can be applied to decompositions concerning many important socio-demographic variables with small

numbers of categories such as sex, cause of death by broad diagnostic groups, education or socioeconomic status by a few aggregate categories, birth order from 1 to 5+ and others. However, there is an obvious difficulty when it comes to the age variable, which has a large number of categories: about 20 and 100 in abridged and complete life tables, respectively, and about 30 in the fertility table. Therefore, it has been suggested to always compute the age components by running the replacement sequence in ascending age order (Andreev et al. 2002). Such an approach guarantees that in the case of life expectancy, the stepwise replacement algorithm's results are exactly equal to results of the most used analytical decomposition formulae by Andreev (1982), Arriaga (1984), and Pressat (1985). To date, the algorithm of stepwise replacement has been used in decomposition of life expectancy, the life table Gini coefficient, healthy life expectancy, lifetime disparity, the standard deviation, the total fertility rate and parity progression ratios (Andreev et al. 2002; Shkolnikov et al. 2003, 2011; van Raalte et al. 2010).

While the algorithm led to useful decomposition formulae for a few demographic indices such as life expectancy and healthy life expectancy, for most demographic indices it is either not possible to get an analytical expression for the underlying components or the resulting formulae are overly complicated for their use on empirical data (for example the Gini coefficient and the lifetime disparity $e^\dagger$).

The stepwise replacement algorithm provides a simple solution to the decomposition task and its generalizability makes it an attractive method of decomposition. It can be extended to a variety of life table based indices and to dimensions other than age. Namely, these are causes of death, states of health, population sub-groups and other categories.
