# Supplementary material for: Decomposing Current Mortality Differences Into Initial Differences and Differences in Trends: The Contour Decomposition Method
Source: Demography. 2017 Jul 28;54(4):1579–602. doi: 10.1007/s13524-017-0599-6 (PMC5547192; doi:10.1007/s13524-017-0599-6)
Supplement: Supplementary file 2 — (PDF 408 kb) [file 13524_2017_599_MOESM2_ESM.pdf]

Online Resource 2. Comparison of the two methods

Both the additive change method and the contour replacement method return similar results in cases where the application of the additive change method is correct and the indicator function f can be approximated by a linear function (by the values used in the argument). Below we formally describe this difference. For simplicity, we consider only the path $B \to b \to a \to A$.

First, we compare estimates for the initial component. Let's consider the initial component in the additive change method

$$\Delta_{+ab}^{i} = \frac{1}{2}\left(f\left(\boldsymbol{m}_{ab}^{[i]} + \Delta\boldsymbol{m}_{AB}^{[i-1]}\right) - f\left(\boldsymbol{m}_{ab}^{[i-1]} + \Delta\boldsymbol{m}_{AB}^{[i-1]}\right)\right) + \frac{1}{2}\left(f\left(\boldsymbol{m}_{ab}^{[i]} + \Delta\boldsymbol{m}_{AB}^{[i]}\right) - f\left(\boldsymbol{m}_{ab}^{[i-1]} + \Delta\boldsymbol{m}_{AB}^{[i]}\right)\right), \quad i = 1, \dots, n. \tag{a2.1}$$

The first component of the first term of (a2.1) can presented as follows

$$\begin{aligned} f\left(\boldsymbol{m}_{ab}^{[i]} + \Delta\boldsymbol{m}_{AB}^{[i-1]}\right) &= f\left(m_a^1 + m_A^1 - m_a^1, \dots, m_a^{i-1} + m_A^{i-1} - m_a^{i-1}, m_a^i + m_B^i - m_b^i, m_b^{i+1} + m_B^{i+1} - m_b^{i+1}, \dots, m_a^n + m_B^n - m_b^n\right) \\ &= f\left(m_A^1, \dots, m_A^{i-1}, m_a^i + m_B^i - m_b^i, m_B^{i+1}, \dots, m_B^n\right) \end{aligned} \tag{a2.2}$$

or using the abbreviation introduced for the contour replacement method

$$f\left(\boldsymbol{m}_{ab}^{[i]} + \Delta\boldsymbol{m}_{AB}^{[i-1]}\right) = f\left(m_{A(a+B-b)B}^{[i]}\right). \tag{a2.3}$$

At the same time

$$f\left(\boldsymbol{m}_{ab}^{[i]} + \Delta\boldsymbol{m}_{AB}^{[i]}\right) = f\left(m_a^1 + m_A^1 - m_a^1, \dots, m_a^i + m_A^i - m_a^i, m_b^{i+1} + m_B^{i+1} - m_b^{i}, \dots, m_b^n + m_B^n - m_b^n\right) = f\left(m_A^1, \dots, m_A^i, m_B^{i+1}, \dots, m_B^n\right) = f\left(\boldsymbol{m}_{AB}^i\right). \tag{a2.4}$$

and

$$\begin{aligned} f\left(\boldsymbol{m}_{ab}^{[i-1]} + \Delta\boldsymbol{m}_{AB}^{[i]}\right) &= \\ &= f\left(m_a^1 + m_A^1 - m_a^1, \dots, m_a^{i-1} + m_A^{i-1} - m_a^{i-1}, m_b^i + m_A^i - m_a^i, m_b^{i+1} + m_B^{i+1} - m_b^{i+1}, \dots, m_a^n + m_B^n - m_b^n\right) \\ &= f\left(m_A^1, \dots, m_A^{i-1}, m_b^i + m_A^i - m_a^i, m_B^{i+1}, \dots, m_B^n\right) \\ &= f\left(m_{A(b+A-a)B}^{[i]}\right), \end{aligned} \tag{a2.5}$$

Thus the equation (a2.1) can be re-written as follows

$$\Delta_{+ab}^{i} = \frac{1}{2}\left(f\left(\boldsymbol{m}_{A(a+B-b)B}^{[i]}\right) - f\left(\boldsymbol{m}_{AB}^{[i-1]}\right)\right) + \frac{1}{2}\left(f\left(\boldsymbol{m}_{AB}^{[i]}\right) - f\left(\boldsymbol{m}_{A(b+A-a)B}^{[i]}\right)\right). \tag{a2.6}$$

By contour decomposition the respective initial component is

$$\Delta_{ab|B}^{i} = f\left(\boldsymbol{m}_{A(a)B}^{[i]}\right) - f\left(\boldsymbol{m}_{A(b)B}^{[i]}\right), \quad i = 1, \dots, n. \tag{a2.7}$$

For simplicity first we consider the case of a simple linear function $f(\boldsymbol{m}) = \sum_{i=1}^{n} k_i m(x_i) + c$. Obviously, (a2.7) will be reduced to the difference between the ith component:

$$\Delta_{ab|B}^{i} = k_i\big(m_a(x_i) - m_b(x_i)\big). \tag{a2.8}$$

Taking into account (a2.6) we get identical result for the initial component calculated using additive changes method

$$\begin{aligned}\Delta_{+ab}^{i} &= \frac{1}{2}k_i\big(m_a(x_i) + m_B(x_i) - m_b(x_i) - m_B(x_i)\big) \\ &\qquad + \frac{1}{2}k_i\big(m_A(x_i) - m_b(x_i) - m_A(x_i) + m_a(x_i)\big) \\ &\qquad = k_i\big(m_a(x_i) - m_b(x_i)\big).\end{aligned} \tag{a2.9}$$

Thus the claim

$$\Delta_{+ab}^{i} \approx \Delta_{ab|B}^{i} \tag{a2.10}$$

is true when $f(\boldsymbol{m})$ can be approximated by a linear function. Moreover, if $f(\boldsymbol{m})$ is differentiable then using a Taylor series we can estimate changes in the target indicator due to changes in the ith element as follows

$$f\left(\boldsymbol{m}_{A(a)A}^{[i]}\right) = f(\boldsymbol{m}_A) + \frac{\partial f}{\partial m_A(x_i)}\big(m_A(x_i) - m_a(x_i)\big) + o\big(m_A(x_i) - m_a(x_i)\big). \tag{a2.11}$$

For the contour replacement method from (a2.8) and (a2.11) we get

$$\begin{aligned}\Delta_{ab|B}^{i} &= f\left(\boldsymbol{m}_{AB}^{[i]}\right) + \frac{\partial f}{\partial m_A(x_i)}\big(m_A(x_i) - m_a(x_i)\big) - f\left(\boldsymbol{m}_{AB}^{[i]}\right) \\ &\qquad - \frac{\partial f}{\partial m_A(x_i)}\big(m_A(x_i) - m_b(x_i)\big) + o\big(m_A(x_i) - m_a(x_i)\big) \\ &\qquad + o\big(m_A(x_i) - m_b(x_i)\big) \\ &\qquad = \frac{\partial f}{\partial m_A(x_i)}\big(m_a(x_i) - m_b(x_i)\big) + o\big(m_A(x_i) - m_a(x_i)\big) \\ &\qquad + o\big(m_A(x_i) - m_b(x_i)\big)\end{aligned} \tag{a2.12}$$

A similar calculation for the additive change method transforms the equation (a2.7) into

$$\begin{aligned}\Delta_{+ab}^{i} &= \frac{\partial f}{\partial m_A(x_i)}\big(m_a(x_i) - m_b(x_i)\big) \\ &\qquad + o\big(m_A(x_i) - m_a(x_i) - m_B(x_i) + m_b(x_i)\big) \\ &\qquad + o\big(m_a(x_i) - m_b(x_i)\big)\end{aligned} \tag{a2.13}$$

Thus

$$\Delta_{+ab}^{i} - \Delta_{ab|B}^{i} \approx o\big(m_A(x_i), m_B(x_i), m_a(x_i), m_b(x_i)\big) \tag{a2.14}$$

A similar calculation for the trend component leads to

$$\delta_{+ab}^{i} - (\delta_{aA|B}^{i} + \delta_{bB|B}^{i}) \approx o\big(m_A(x_i), m_B(x_i), m_a(x_i), m_b(x_i)\big). \tag{a2.15}$$

Here it makes sense to note that formulae for a linear function *f* similar to (a2.6) and (a2.7) for the trend component has the form

$$\delta_{+ab}^{i} = \frac{1}{2}\left(f\left(\boldsymbol{m}_{A(b+A-a)B}^{[i]}\right) - f\left(\boldsymbol{m}_{AB}^{[i-1]}\right)\right) + \frac{1}{2}\left(f\left(\boldsymbol{m}_{AB}^{[i]}\right) - f\left(\boldsymbol{m}_{A(a+B-b)B}^{[i]}\right)\right) = m_A(x_i) - m_a(x_i) + m_b(x_i) - m_B(x_i) \quad \text{(a2.16)}$$

and

$$\delta_{ab|AB}^{i} = \delta_{aA|B}^{i} + \delta_{bB|B}^{i} = f\left(\boldsymbol{m}_{AB}^{[i]}\right) - f\left(\boldsymbol{m}_{A(a)B}^{[i]}\right) + f\left(\boldsymbol{m}_{A(b)B}^{[i]}\right) - f\left(\boldsymbol{m}_{AB}^{[i-1]}\right) = m_A(x_i) - m_a(x_i) + m_b(x_i) - m_B(x_i), \quad \text{(a2.17)}$$

respectively.

In conclusion, if the demographic indicator is not too sensitive to changes in the elementary components of a vector $\boldsymbol{m}$ and can be represented as

$$f\left(\boldsymbol{m}_{A(a)A}^{[i]}\right) = f(\boldsymbol{m}_A) + \phi\big(m_A(x_i) - m_a(x_i)\big), \quad \text{(a2.18)}$$

where $\phi(x) \to 0$ by $x \to 0$, differences between components of vectors $\boldsymbol{m}_A, \boldsymbol{m}_B, \boldsymbol{m}_a$, and $\boldsymbol{m}_b$ are relatively small, then both methods return similar results.
