# Supplementary material for: Decomposing Current Mortality Differences Into Initial Differences and Differences in Trends: The Contour Decomposition Method
Source: Demography. 2017 Jul 28;54(4):1579–602. doi: 10.1007/s13524-017-0599-6 (PMC5547192; doi:10.1007/s13524-017-0599-6)
Supplement: Supplementary file 4 — (PDF 517 kb) [file 13524_2017_599_MOESM4_ESM.pdf]

## Online Resource 4. Empirical comparison of the additive component and the contour replacement methods using the HMD data

We compared the two approaches on aggregate life table indices using data from the Human Mortality Database (2016). Pairwise comparisons were done between all countries 10, 20 and 30 years apart from the first year of each decade for each sex, using single year of age and single calendar year (1x1) and 5-year age categories and single calendar year (5x1) life tables. The full list of life tables used is given in table a4.1.

| Country | First year | Last year |
|---|---|---|
| Australia | 1930 | 2000 |
| Austria | 1950 | 2010 |
| Belarus | 1960 | 2010 |
| Belgium | 1950 | 2010 |
| Bulgaria | 1950 | 2010 |
| Canada | 1930 | 2000 |
| Czech Republic | 1950 | 2010 |
| Denmark | 1850 | 2010 |
| East(-ern) Germany | 1960 | 2010 |
| England & Wales | 1850 | 2010 |
| Estonia | 1960 | 2010 |
| Finland | 1880 | 2000 |
| France | 1930 | 2010 |
| Hungary | 1950 | 2000 |
| Ireland | 1950 | 2000 |
| Italy | 1880 | 2000 |
| Japan | 1950 | 2010 |
| Latvia | 1960 | 2010 |
| Lithuania | 1960 | 2010 |
| Netherlands | 1850 | 2000 |
| New Zealand* | 1950 | 2000 |
| Northern Ireland | 1930 | 2010 |
| Norway | 1850 | 2000 |
| Poland | 1960 | 2000 |
| Portugal | 1940 | 2010 |
| Russia | 1960 | 2010 |
| Scotland | 1860 | 2010 |
| Slovakia | 1950 | 2000 |
| Spain | 1910 | 2010 |
| Sweden | 1850 | 2010 |
| Switzerland | 1880 | 2010 |
| Taiwan | 1970 | 2010 |

| | | |
|---|---|---|
| Ukraine | 1960 | 2000 |
| United States of America | 1940 | 2010 |
| West Germany | 1960 | 2010 |

**Table a4.1** HMD life table data used for comparison of the additive component and the contour replacement methods.

We compared components for 6 different indices of central tendency and variation: life expectancy, the median age at death, lifetime disparity ($e$†), the Theil index, the average inter-individual difference, and the standard deviation (Table a4.2).

| Index | Formulae, discrete life table data |
|---|---|
| Median age at death | $\hat{x}\ such\ that\ \ell(x) = 0.5$ |
| Lifetime disparity | $\sum_{x=0}^{\omega-1} d(x)\bar{e}(x)$ |
| Theil Index | $\sum_{x=0}^{\omega-1} d(x)\left(\frac{\bar{x}}{e(0)}\ln\frac{\bar{x}}{e(0)}\right)$ |
| Average inter-individual difference | $\left[1 - \frac{1}{e(0)}\sum_{x=0}^{\omega-1}\left(\bar{\ell}(x)\right)^2\right]e(0)$ |
| Standard deviation | $\sqrt{\sum_{x=0}^{\omega-1} d(x)(\bar{x} - e(0))^2}$ |

**Table a4.2** Definitions of functions used for comparison of the additive change and the contour replacement methods. Note: in all formulae, it is assumed that $l(0)$ = 1.

All indices were calculated from discrete formulas for life tables with a radix of $\ell(0)$=1. In the formulas above, $\ell(x)$ and $e(x)$ are respectively the number of survivors and remaining life expectancy at age $x$, and $d(x)$ is the number of deaths over ages $x$ to $x + 1$. An overbar denotes the average life table quantity over the age interval $x$ to $x + 1$. Thus for instance, $\bar{e}(x) = e(x) + a(x)(e(x + 1) - e(x))$, where $a(x)$ is the proportion of the age interval lived by those who died. The oldest age in the life table is denoted by $\omega$. The median age at death was calculated by fitting a spline through the $\ell(x)$ curve to estimate the age (to 2 decimals) at which half of the life table cohort had died.

In Fig. a4.1 we show the correspondence between the initial age contributions estimated from the two decomposition methods using 1x1 life tables of the HMD, 10 years apart. Removing the decompositions with negative $m(x)$ values produced at any age left us with 261,183 initial age components from 2,353 decompositions.

The two methods produced very similar results for life expectancy, lifetime disparity, the Theil index, and the average inter-individual difference between ages at death. The initial components of the standard deviation decompositions were also similar, however the contour method produced higher estimates when the age contribution was strongly positive, and lower estimates when the age contribution was strongly negative. These results are understandable in light of condition (28). Indeed, the standard deviation has a much higher sensitivity to small changes in the argument ("aversion to inequality" according to Anand et al. (2002)) compared to the mean, the average inter-individual difference or the Theil index.

The initial components estimated for the median age at death also fell along the diagonal line of equality between methods, however with more noise than for life expectancy and indices of variation. This is unsurprising given that it is a fitted index instead of a calculated index—we fitted a spline through the survivorship curve to estimate the age at which half of the life table cohort had died.

The modal age at death (not shown in Fig. a4.1) is another life table index that is problematic with respect to comparability of the contour and the additive change decomposition methods. The mode is not a differentiable function of age-specific death rates and can experience very large changes when in its extreme the maximum number of deaths jumps from age 0 to an older adult age. Even the adult modal age at death can jump around between two or more adult ages, especially when mortality is not smoothed over age.

The associations between trend components produced by the two decomposition methods (not pictured) were similar to those presented for the initial components. This is obvious given that the initial and trend components sum to the final components, and the final components are exactly equal for the two decomposition methods.

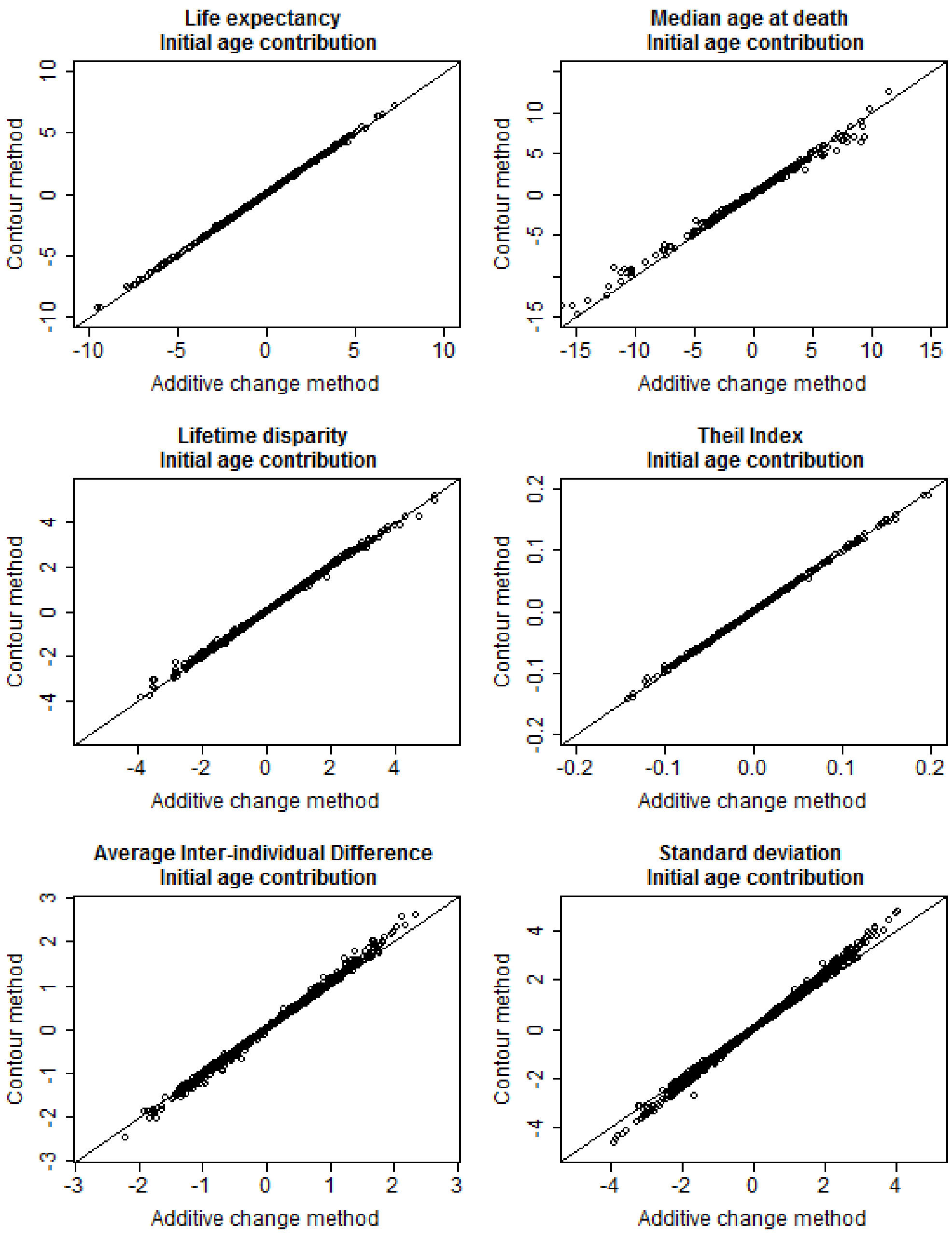


**Fig. a4.1** A comparison of the initial conditions component determined by the contour and additive change decomposition methods, on 2,353 male and female 1x1 life tables of the HMD, 10-years apart (261,183 individual age components).

Finally for two of the indices, life expectancy and lifetime disparity, we fitted a linear regression to the initial conditions' components estimated for the two methods (Table a4.3). For both indices, the estimates were close to 1 in all cases with low standard errors. This gives us confidence that the methods can be used interchangeably in cases where there is no negative *m*(*x*) produced in the additive change method.

| | | Life expectancy | | Lifetime disparity | |
|---|---|---|---|---|---|
| Age x Year | $T - t$ interval | **Estimate** | **Standard Error** | **Estimate** | **Standard Error** |
| 1x1 | 10 years | 1.000 | 0.00003 | 1.003 | 0.00008 |
| 1x1 | 20 years | 0.991 | 0.00009 | 0.973 | 0.00023 |
| 1x1 | 30 years | 0.979 | 0.00020 | 0.941 | 0.00048 |
| 5x1 | 10 years | 1.009 | 0.00006 | 1.010 | 0.00022 |
| 5x1 | 20 years | 1.015 | 0.00014 | 1.008 | 0.00046 |
| 5x1 | 30 years | 1.020 | 0.00032 | 0.998 | 0.00092 |

**Table a4.3** Linear regression results with the initial age components of the additive method used as predictor and the initial age components of the contour method as response variables.

A total of 261,183 initial age components were compared by means of 2,353 regressions based on 2,353 decompositions. All estimates have the significance level $p \leq .001$.
